# Assessment of Crop Damage by Protected Wild Mammalian Herbivores on the Western Boundary of Tadoba-Andhari Tiger Reserve (TATR), Central India

**DOI:** 10.1371/journal.pone.0153854

**Published:** 2016-04-19

**Authors:** Abhijeet Bayani, Dilip Tiwade, Ashok Dongre, Aravind P. Dongre, Rasika Phatak, Milind Watve

**Affiliations:** 1 Indian Institute of Science Education and Research (IISER), Pune, Dr. Homi Bhabha Road, Pashan, Pune 411008, Maharashtra, India; 2 Department of Biodiversity, Abasaheb Garaware College, Pune, India; Louisiana State University & LSU AgCenter, UNITED STATES

## Abstract

Crop raiding by wild herbivores close to an area of protected wildlife is a serious problem that can potentially undermine conservation efforts. Since there is orders of magnitude difference between farmers’ perception of damage and the compensation given by the government, an objective and realistic estimate of damage was found essential. We employed four different approaches to estimate the extent of and patterns in crop damage by wild herbivores along the western boundary of Tadoba-Andhari Tiger Reserve in the state of Maharashtra, central India. These approaches highlight different aspects of the problem but converge on an estimated damage of over 50% for the fields adjacent to the forest, gradually reducing in intensity with distance. We found that the visual damage assessment method currently employed by the government for paying compensation to farmers was uncorrelated to and grossly underestimated actual damage. The findings necessitate a radical rethinking of policies to assess, mitigate as well as compensate for crop damage caused by protected wildlife species.

## Introduction

Agricultural lands close to protected areas (PAs) often face crop raiding by wild herbivores, which can be a serious problem for farmers whose livelihoods depend on agricultural produce [1–4]. In order to avoid economic loss, farmers apply a range of protective measures. They include manual guarding, various types of fences, trenches and other devices [5–13]. However, these measures often come with high associated costs [14] and risks [11, 15–19]. The traditional fences are made using wooden poles and thorny branches lopped from nearby forests causing substantial damage to the forest. Destructive measures such as traps can kill or injure animals. Highly sophisticated means such as electric fences are expensive and need continued maintenance [14, 20]. Although a number of measures have been developed and shown to be effective on an experimental scale, there are reason why they achieve limited success when employed on a wider spatial scale (Watve et al, manuscript under review).

Economic loss due to wildlife is a considerable threat to animal conservation due to increasing resentment among the residents that may result into retaliation [21–27]. Appropriate compensation is thought to reduce conflict, making conservation efforts more effective [27, 28]. At least in one case, that of wolves in Yellowstone national park, compensation was shown to be an effective conservation policy [27]. Even if we make no assumption of compensation helping conservation, from social justice point of view the government may accept it as its duty to compensate farmers’ loss. In many countries, including our study area, the laws enable compensation of damage to the suffering farmers [26]. Although a number of studies on crop raiding are published addressing the problem in different habitats and caused by different species of wild animals, few utilize rigorous methods for primary estimation of damage and attempt to cross check or validate the methods [4, 29–32]. Some rigorous methods for damage estimation are suggested in the context of rodent damage [33] which are highly man-power intensive and no such methods have been used in compensation protocols in the study area. Since, the legal protocols in our study area have no clear guidelines on how to estimate the extent of damage, a visual inspection and assessment of damage is made accompanied by negotiations between the farmer and the compensating authority. This leads to a subtle ongoing conflict between farmers and park officials. It also brings about a change in perception of the farmers. Animals that were once perceived to be a part of nature are now perceived as a property of the park and a cause of menace to them. This “your animal syndrome” is likely to be more injurious to conservation in the long run than the actual damage to crops and the compensation paid [34].

Patterns of damages caused by different herbivores can be substantially different and estimating them using a single method may not be possible. For example, raiding by Asian elephant (*Elephas maximus*) and African elephant (*Loxodonta africana*) leads to visibly obvious damage over a measurable area whereas smaller to medium sized herbivores like, blackbuck (*Antilope cervicapra*), nilgai (*Boselaphus tragocamelus*), chital (*Axis axis*), wild pig (*Sus scrofa*) etc. may chew or nibble some specific parts of a plant such that the damage is not obvious at a glance [27, 35] but yields can be affected significantly [1].

Even if we assume that there is some way of accurately estimating the damage during an inspection following the raid, there are more complications. The crop species are also living entities that respond to inflicted damages in an adaptable manner. If the damage is not lethal to a plant, it regrows and tries to make up for the loss at least partly. Thus, the net damage at the end of the season may be substantially different from what appears immediately after a raid. One study that addressed this question showed that the visible damage was not correlated well with the grain yield at harvest [36].

Government records show that between 0.1 to 8% farmers received compensation during the years 2009 to 2015. This was in contrast with farmers’ perception that over 90% farmers in the buffer zone suffered some loss. The farmers that received compensation, claimed that not more than 20% of the actual loss was compensated (Bayani et al, manuscript under preparation). Out study was motivated mainly by this difference. We used in this study, four different methods of damage estimation in the study area to address the question, whether the farmers’ perception was more realistic or the compensation records of the government, or both were biased in different ways. Since different methods of damage estimation have different sources of errors and biases, if they converge on a similar inference, the inference can be more reliable. If they do not converge, a comparison would show whether some of them give consistent under or overestimates as compared to others [37–38]. This can be used to choose appropriate methods towards offering realistic compensation in near future.

## Study Area

**Fig 1:**
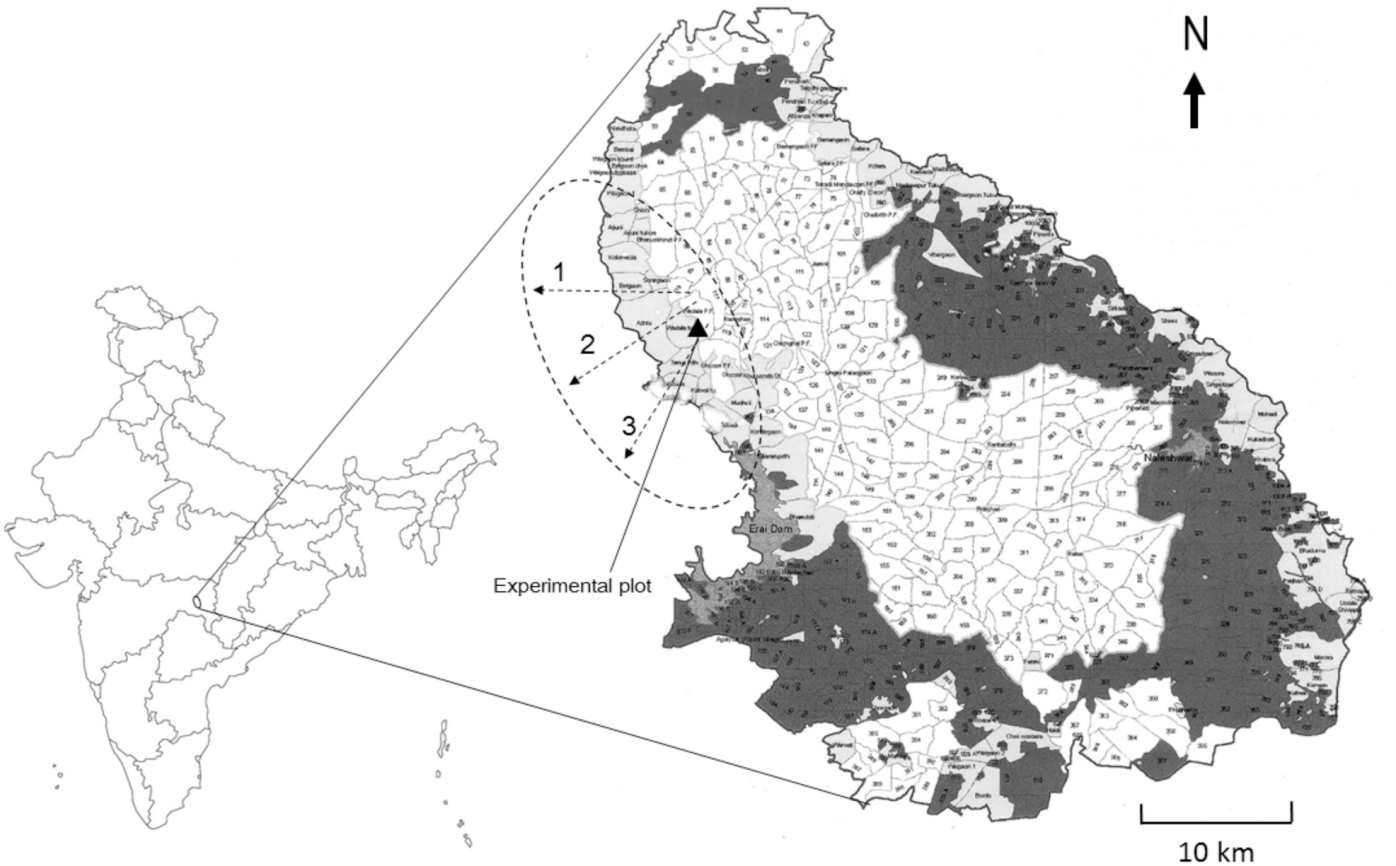
Map and location of study area. Light gray shaded zones denote villages and dark gray denote Division Forest area; both together constituting the buffer zone. The buffer area comprises over 70 villages with agriculture as the main livelihood. The dotted ellipse represents our study area. Location of the experimental plots is indicated by the dark triangle and the three transect lines extending from forest boundary into agricultural lands are shown by dotted arrows.

The Tadoba-Andhari Tiger Reserve (TATR, 19° 59’–20° 29’ N and 79° 11’–79° 40’ E) is located in Chandrapur district of Maharashtra, India. The Tiger Reserve extends over 1727 sq. km out of which 625.5 sq. km is the core zone (Fig 1). TATR is a Teak (*Tectona grandis*) dominated mixed forest of deciduous trees including *Diospyros melanoxylon*, *Terminalia elliptica*, *Butea monosperma*, *Chloroxylon sweitenia* and bamboo (*Dendrocalamus* sp. and *Bambusa* sp.), supporting good faunal diversity. We selected the western boundary buffer (of the core) where through most of the length, the transition between forest cover and agriculture lands creates a sharp ecotone. Only in certain areas outside the western boundary, there is a mosaic of agricultural lands and forest patches. Crops are cultivated in two seasons, *viz*. kharif (monsoon crops) and rabi (winter crops). Rice (*Oryza sativa*) and soybean (*Glycine max*) are the primary kharif crops whereas wheat (*Triticum aestivum*) and chickpea (*Cicer arietinum*) are primary rabi crops. We selected these four crop species for all of our observations and experiments as they are the most abundant crops in the study area. Apart from these, cotton (*Gossypium arboreum*), turmeric (*Curcuma longa*), flax or linseed (*Linum usitatissimum*), and grass pea or sweet blue pea (*Lathyrus sativa*) are other secondary crops taken in comparatively lesser extent. The mammalian fauna of the western periphery of TATR is dominated by herbivore species including nilgai (*B*. *tragocamelus*), chital or spotted deer (*A*. *axis*), wild pig (*S*. *scrofa*) and carnivore species including tiger (*Panthera tigris*), leopard (*Panthera pardus*), dhole (*Cuon alpinus*) and sloth bear (*Melursus ursinus*).

## Materials and Methods

We used four independent methods to directly or indirectly estimate crop damage. (1) periodic visual examination of crop damage along transect lines going away from forest boundary; (2) the net grain yield per unit area along the same transect lines, measured at the time of harvest; (3) comparison of yields on protected and exposed neighboring farms; (4) comparison of grain yields after controlled artificial herbivory. Across the four methods one or more of the three parameters were monitored namely, (i) Frequency of visits by wild herbivores (ii) Visual estimate of apparent damage, and (iii) Grain yield at harvest, all the three were estimated taking an individual farmer’s cultivated farm as a unit and then normalized by the area under cultivation in that farm. We report the results of 6 years of study form 2009 to 2015 in this paper. Owing to manpower limitations, each of the four methods could not be employed across all the six years but we ensured that each method was replicated sufficiently to ensure reproducibility (Table 1). Whenever analysis compared the results of two or more of the methods, the comparison was made in the same season and same area.

**Table 1:** Temporal overlap of methods.

Year	Methods used
2009–2012	Method-1, 2
2012–2013	Method-2
2013–2015	Method-2, 3, 4

### 1. Visual estimates of frequency of raids and area damaged

Three transect lines each 10 km long were laid going away from the boundary of the core area of TATR. Since there was no forest cover available to animals outside this boundary, we expected the raiding frequency to be a monotonic decreasing function of distance from forest. Geographical location of the center of each farm that was cut by the line was recorded using handheld GPS device (Garmin 60). Baseline information about the owners and the cropping season, crop species, total area of farm, area under cultivation of each crop, irrigation facility and other agriculture related information was noted. A total of 137 farms along the transects were then visited once every week by our research personnel during daytime to observe whether there were visible areas of damage. Whenever damage was noted, the approximate area with visible damage was measured in meter squares. This mimics the currently employed method of visual inspection to estimate damage. The weekly observations continued until the crop was harvested. This information was treated as binary to calculate per day probability of damage assuming that the raids were random and therefore followed Poisson distribution. Since a visible damage would mean one or more events of damage, *P*_*1*_, *P*_*2*_ *… P*_*n*_ from the Poisson series could not be estimated empirically. But, since no crop raiding meant no damage, an empirical estimate of *P*_*0*_ was possible. Using Poisson formula for *P*_*0*_ *= 1/ e* ^*μ*^, the mean number of raids per week (*μ*) could be calculated, which when divided by 7 gave the mean frequency of damaging raids per night.

### 2. Grain yield at harvest

The farms along the transect lines up to 6 km mentioned above were visited at the time of harvest to note the total grain yield for each crop per unit area. Since the harvesting operations were at various stages at the time of visit by research personnel, the actual yield was not directly accessible for inspection every time. However, in at least 20% of cases, the research personnel could physically verify the grain yield at the time of harvest in terms of number of bags or at the time of sale in terms of actual weight in quintals. We studied the farms along transects for subsequent years and recorded yields at 180 farms for both kharif and rabi season. Grain yield was normalized with individual farmer’s land area under cultivation and expressed as quintals per hectare (Q/Ha)

### 3. Experimental plots

A plot of approximately one hectare at close proximity to forest was used as an experimental farm. This farm was the first from the forest along one of the transect lines. The experimental area with homogeneous soil and irrigation conditions was divided into four sub-plots two of which were fenced with a combination of barbed wire and thorny bush and the other two left unprotected. Four crop species namely, rice and soybean during kharif and wheat and chickpea during rabi season were grown in neighboring protected and unprotected farms keeping the parameters of cultivation such as soil preparation, fertilizer use, seed density and irrigation identical. All the experimental farms were protected during daytime to avoid any damage by domestic animals and were observed silently at night from traditionally prepared 10–12 ft tall wooden watchtowers or guarding platforms, locally termed as ‘*mara*’ or ‘*machan’*. The daily-recorded parameters included frequency of visits by wild herbivores, their group size, frequency of visible damage and area with visible damage. At the end of the season, the grain yield on harvest per unit area was recorded.

### 4. Artificial herbivory

To study the effect of levels of damage on individual plants, particularly their regrowth after damage and the resultant grain yield, the plants were manually cut using scissors at different heights and different ages and compared with uncut control plants at the time of harvest. These experiments were performed in a fenced area independent of method 3. Three species namely soybean, chickpea and wheat were subject to these experiments during two consecutive seasons of 2013 and 2014. In one set of experiments, the main stems of all plants in a unit sampling area were cut at different heights from ground in a pre-flowering stage (at 60 days for wheat, 55 days for soybean and chickpea). In another set of experiments the tips comprising leaves and buds in the upper 2–3 cm were cut at different ages of the crop (see Table 2). The plants were allowed to regrow through rest of the season. All the treatment plots of all crop species were provided with the same amount and combination of fertilizers, pesticides, and water as the control plots. At the time of harvest, all the treatment and control plants were uprooted carefully to measure the different parameters such as the height of the regrown plant, canopy height and width (for chickpea only), number of branches (for soybean and chickpea), the number of pods/heads and number of grains/seeds (for all the three species).

**Table 2:** Experimental design for artificial herbivory of wheat, soybean and chickpea.

Crop species	Plot area (sq. m.)	Height at which plants cut (cm)	Number of plants (n)	Age at which plants cut (days)	Number of plants (n)
Wheat	1	Control	125	Control	125
	1	5	176	25	92
	1	10	178	45	202
	1	15	205	55	199
Soybean	1	Control	108	Control	108
	1	5	125	20	87
	1	10	128	45	107
	1	15	100		
	1	20	74		
Chickpea	2	Control	50	Control	50
	2	5	50	25	53
	2	10	51	45	51
	2	15	54		

## Results

### 1. Periodic monitoring of farms along transects

**Fig 2:**
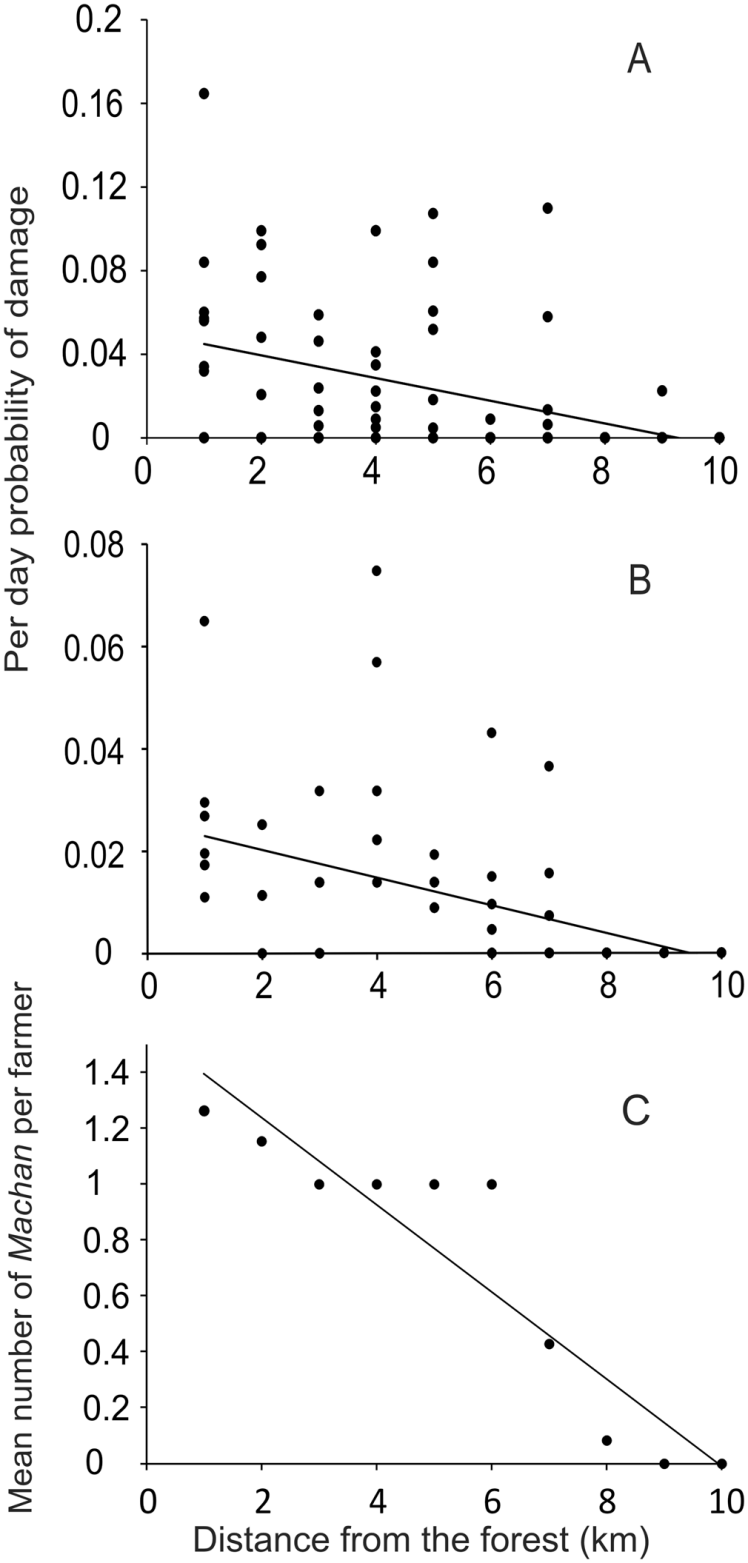
Trend of per day probability of damage pooled from three transect lines. For each of the observed weeks, per day Poisson probability of raid between every one kilometer interval was calculated from the fraction of undamaged farms from all the three transects. A: Trend in kharif season (r = -0.4525, p = 0.0001, n = 90); B: Trend in rabi season (r = -0.5455, p = 0.0001, n = 98). C: Trend in average number of *machans* per farm along the transects (r = -0.9310, p<0.0001, n = 10).

The mean frequency per night, calculated using Poisson probabilities, showed a decreasing trend with distance from the edge of forest (Fig 2A and 2B). Although both seasons showed a declining trend with distance, the damage frequency in kharif (Fig 2A) was nearly twice that in rabi (Fig 2B) over the 10 km stretch. This difference is likely to be owing to active guarding by farmers, which is difficult during monsoon and therefore not practiced.

It is important to note that the frequency of damage in (Fig 2A and 2B) is in spite of manual guarding efforts. Frequency of animal visits to a farm could be substantially greater than the frequency of inflicting visible damage, as raider animals are often driven away by the vigilant farmers. Consistent with the decreasing trend in the frequency of crop raiding, farmers’ efforts at guarding declined with distance. Fig 2C shows the trend in the mean number of *machans* per farmer at one kilometer intervals along the transect lines. Farmers close to the forest often made more than one *machans* barring which one *machan* per farm was the modal trend. There appeared to be a threshold risk below which it was perhaps not perceived worth making a *machan* since we see a sharp decline in the number after 6 km.

### 2. Grain Yield along transect farms

**Fig 3:**
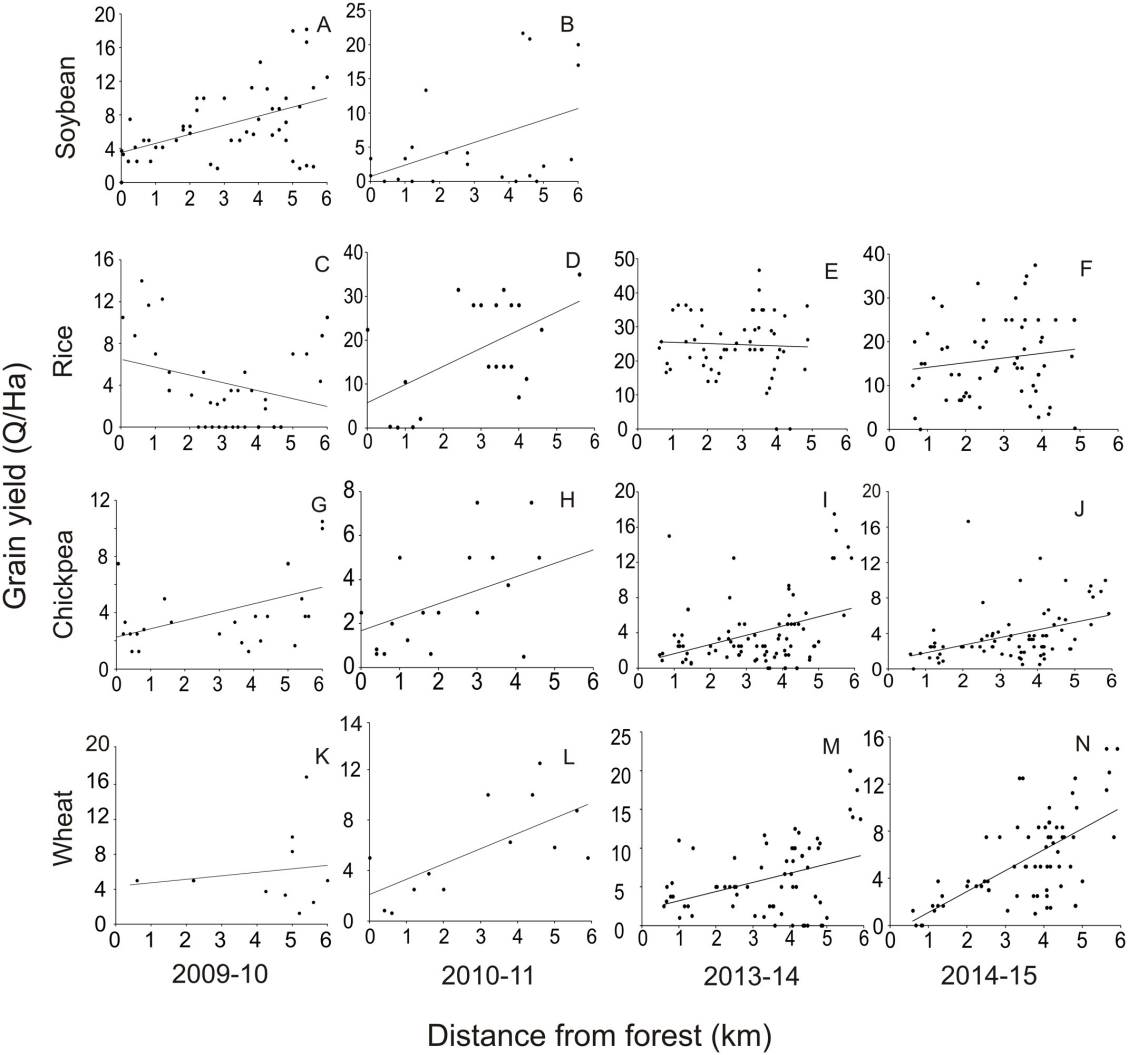
Trend of grain yield at harvest with distance from PA boundary for 4 crops over 4 seasons. Soybean: A 2009 (r = 0.473, p = 0.0001, n = 95) and B 2010 (r = 0.448, p = 0.03, n = 22); Rice: C 2009 (r = -0.291, p = 0.08, n = 35), D 2010 (r = 0.53, p = 0.001, n = 20), E 2013 (r = -0.044, p = 0.73, n = 56) and F 2014 (r = 0.14, p = 0.28, n = 58); Chickpea: G 2009–10 (r = 0.466, p = 0.012, n = 27), H 2010–11 (r = 0.54, p = 0.01, n = 17), I 2013–14 (r = 0.378, p = 0.0029, n = 83) and J 2014–15 (r = 0.398, p = 0.0003, n = 78); Wheat: K 2009–10 (r = 0.147, p = 0.66, n = 10), L 2010–11 (r = 0.67, p = 0.01, n = 12), M 2013014 (r = 0.369, p = 0.004, n = 65) and N 2014–15 (r = 0.642, p = 0.0001, n = 67).

Corresponding to the decreasing trend of visible damage by herbivores, there was an increasing trend in grain yield with distance from the forest boundary along the transects. With the exception of rice, there was a significant and consistent increasing trend with distance for soybean, chickpea and wheat (Fig 3).

The distance trends in rice appear to differ from those in other crops. For all other crops the trends in the yield were consistent with the frequency of herbivore damage. The trend in the frequency of animal raids between the first kilometer and the interval between 5^th^ and 6^th^ km showed about twofold decline in the frequency of raids. Compared to this decline the yield improved by 2.15 to 4.5 fold for soybean, 2.03 to 4.24 fold for chickpea and 1.37 to 2.85 fold for wheat. The trend lines of grain yield also give us a rough estimate of average damage close to the forest. For crops other than rice, the slopes of the trend lines range from 0.4 to 1.78. The average yields at 0–1 km are between 28 to 78% of the average yields at a distance of 5–6 km. This comparison indicates that the yield deficit due to all causes combined close to the PA, range from 28 to 78% for crops other than rice.

**Fig 4:**
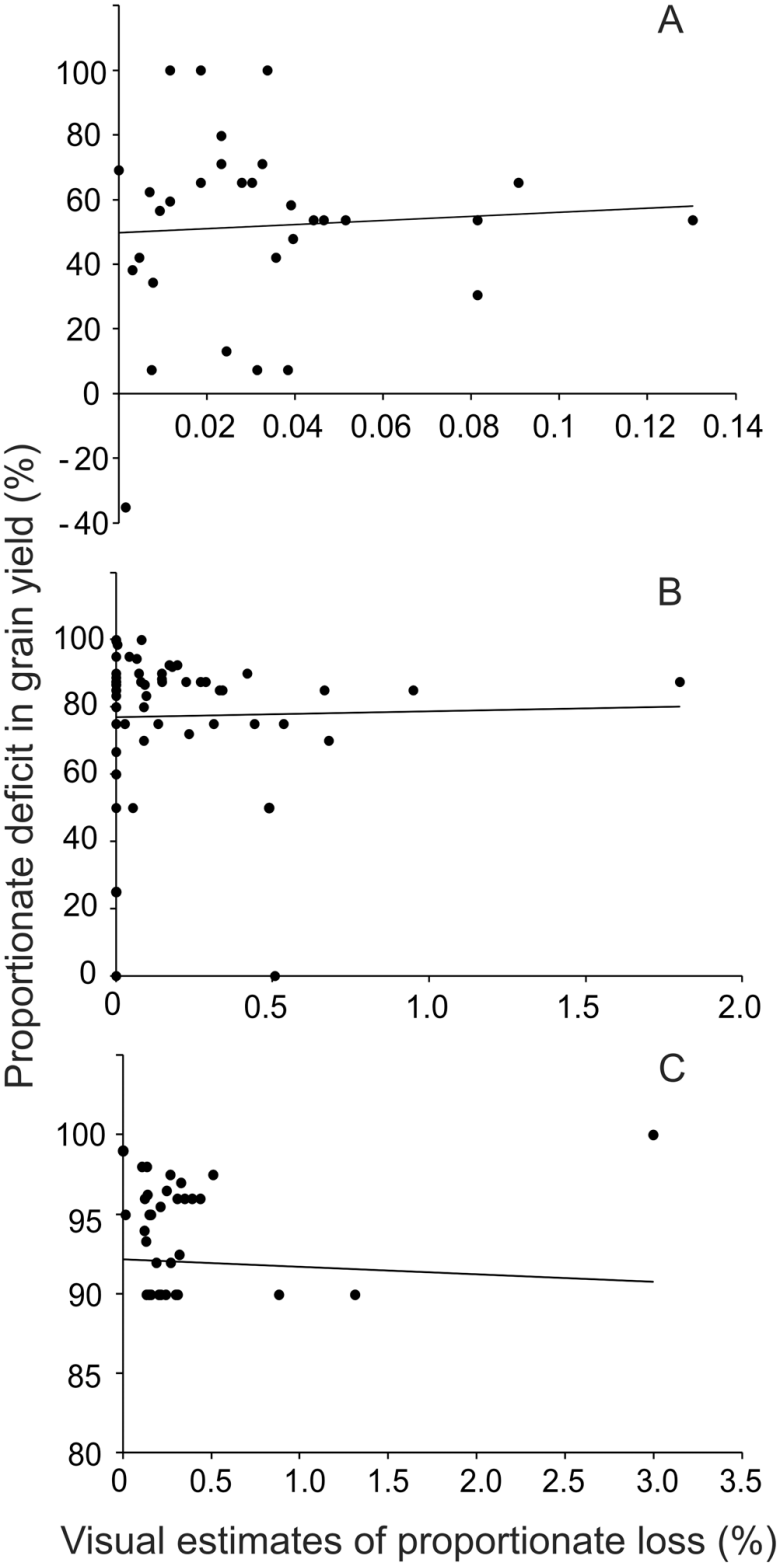
Comparison of visually estimated loss and actual deficit in grain yield at harvest as compared to fenced control plots (both expressed in percentage). A: Rice (r = 0.062, p = 0.73, n = 32), B: Chickpea (r = 0.022, p = 0.86, n = 63), C: Wheat (r = -0.0519, p = 0.75, n = 39). All trends remained non-significant even after removing outliers. Apart from lack of correlation, note the orders of magnitude difference in scales. Cumulative visual assessment was dramatically lower than yield deficit.

A comparison of grain yields with the visual estimates of the area damaged made during weekly visits to the farms, revealed a poor correlation between visually estimated damage and the reduction in net yield from the expected (Fig 4). For this analysis done on four seasons’ (2009–2011) data, a cumulative of the weekly visual estimate of damage was correlated with the deficit from expected yield. The expected was taken to be the average yield at a distance between 5 to 6 km for a given crop and given season. All the correlations were non-significant and throughout the range, the deficit in grain yield was orders of magnitude greater than the cumulative visual estimate of damage.

### 3. Experimental farms

Frequency of damage could be measured for four crops separately on experimental farms. We observed that rice did not face severe raiding problems before seed setting, whereas wheat faced raiding at all stages except after seed setting. Post-harvest raiding was prevalent in rice stacks but not for wheat. Soybean and chickpea were susceptible throughout the season.

**Fig 5:**
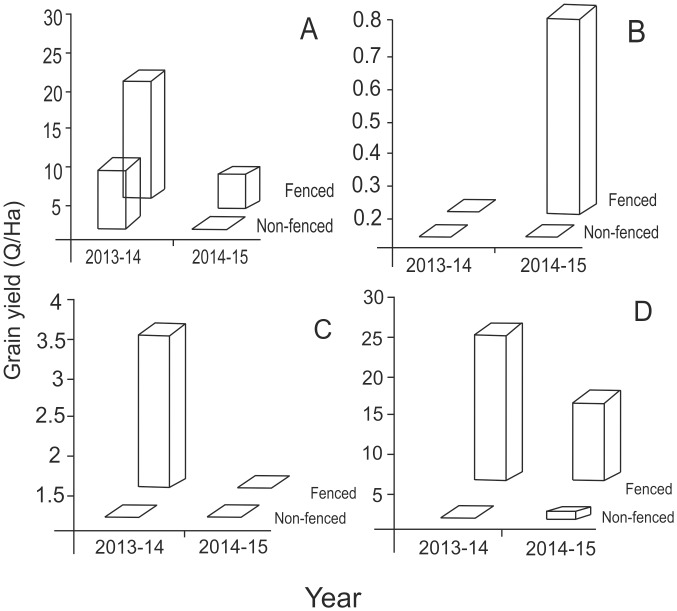
Comparison of grain yield at harvest in fenced and non-fenced plots for 4 crops in two seasons. A: rice, B: soybean, C: chickpea, D: wheat. Soybean in 2013–14 and chickpea in 2014–15 failed due to reasons other than herbivory.

In all four experimental crops cultivated over two seasons, the non-fenced plots faced severe damage due to herbivory compared to the fenced plots. The fenced plots were not completely protected. Indian hare (*Lepus nigricollis*) were observed to make their way through the fence frequently. Nilgai, chital and wild pigs demonstrated their ability to negotiate the fence on occasions although the frequency of their visits to fenced and unfenced areas was substantially different. Most instances of entering the fenced areas were after the crops on the neighboring unfenced areas were almost completely devoured. Grain harvest at the end of the season revealed that wheat, soybean and chickpea faced 100% loss in the unprotected and unguarded farms. Rice was least damaged but still faced a 68% loss in the unfenced unguarded areas in 2013. In 2014, owing to unfavorable rainfall pattern accompanied by a disease, the overall rice crop suffered substantially. In this season, the unprotected area yielded nil, whereas the protected area yielded 7.68 Q/Ha (Fig 5).

### 4. Artificial herbivory

Since crops are living entities, partially damaged plants can regrow [33, 39]. Plants can also show life history trade-offs on facing challenge of herbivory [39]. Therefore, a realistic estimation of damage should also account for recovery by compensatory growth and altered life history traits if any. Artificial herbivory experiments by cutting the shoot tips at measured heights or at certain age of plants revealed that there was substantial growth after cutting. Nevertheless, there appeared to be a cost associated with compensatory growth reflected in deficient grain yield.

**Fig 6:**
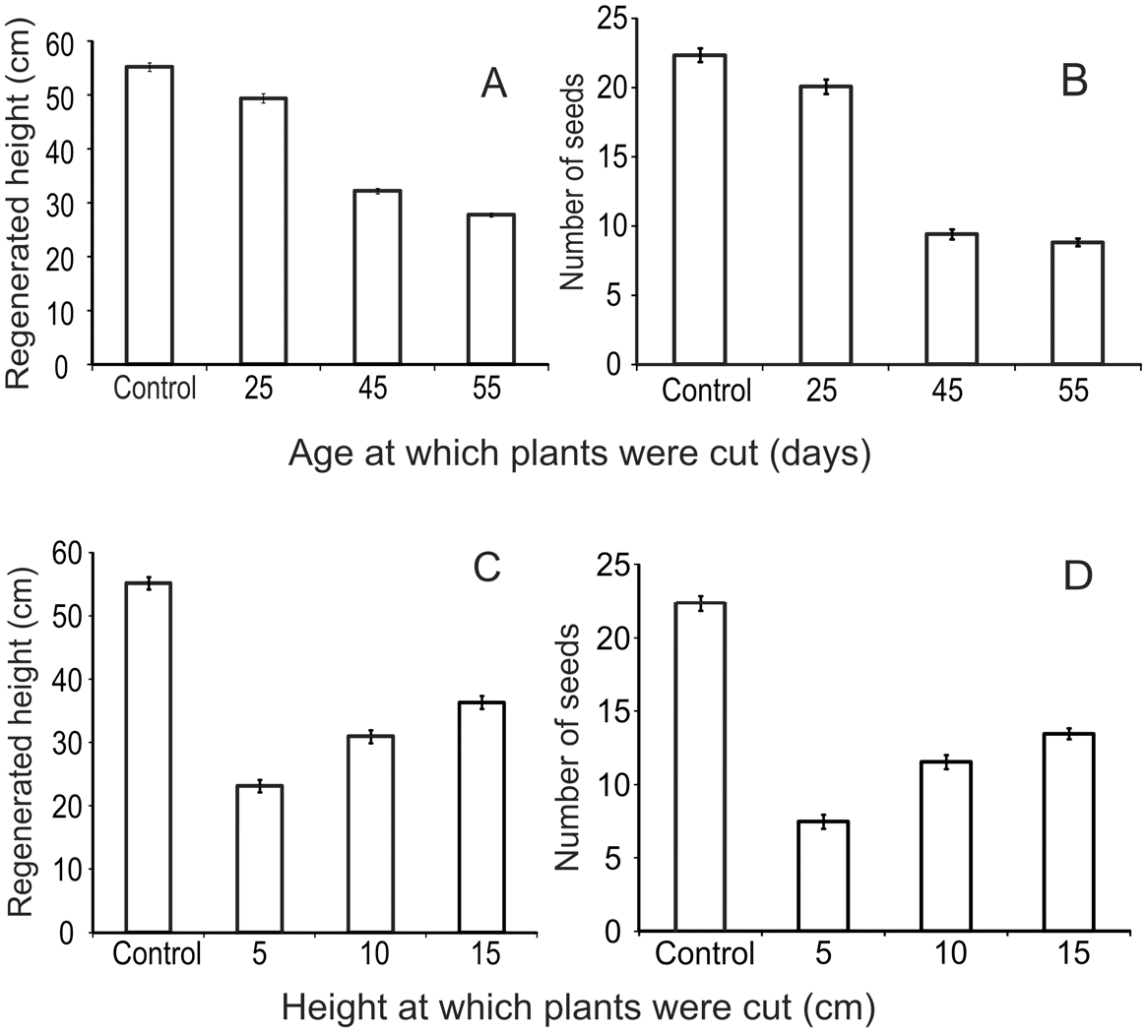
Artificial herbivory in Wheat: comparison of regrowth by wheat plants cut at different age. A: vegetative regrowth, B: number of seeds after regrowth (control, n = 125; age 25, n = 92; age 45, n = 202; age 55, n = 199) and comparison of regrowth of vegetative part in wheat plants cut at various heights at pre-flowering stage: C: vegetative regrowth, D: number of seeds. (Control, n = 125; height 5, n = 176; height 10, n = 178; height 15, n = 205).

In wheat, we observed that plants cut at the age of 25 days from sowing regrew substantially and gained a height comparable with the control at harvest. The grain yield was also comparable to the controls (Fig 6A & 6B). However, when cut at later ages it did not recover sufficiently in height as well as seed number. In other words, early damage appeared to allow greater time for regrowth resulting into better grain yield. If cut after the flowering stage, there was no seed formation. Thus in wheat damage at later stages of crop appeared to be more serious. When groups of plants were cut at different heights in a pre-flowering stage they recovered partially in terms of height and produced some seed but the yield was substantially lower, the deficit in yield being proportional to the extent of cutting (Fig 6C & 6D).

**Fig 7:**
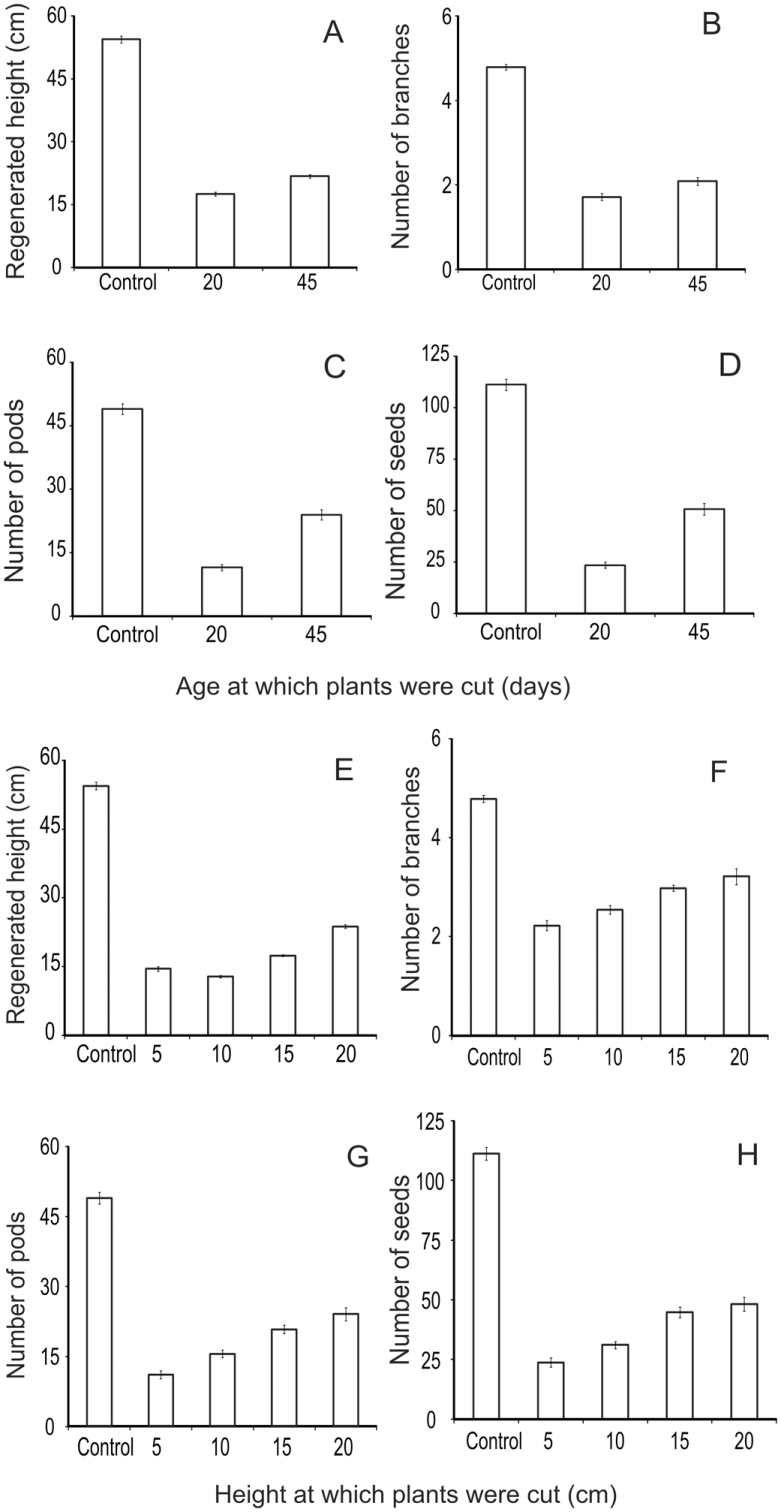
Regrowth after artificial herbivory in soybean at different ages. A: regenerated height, B: number of branches, C: number of pods and D: number of seeds 20 days (n = 87) and 45 days (n = 107) with control (n = 108). Regrowth after artificial herbivory at different heights in pre-flowering stage in soybean. E: regenerated height, F: number of branches, G: number of pods, H: number of seeds in plants cut at 5 (n = 125), 10 (n = 128), 15 (n = 100), 20 (n = 74) with control (n = 108).

In soybean, the age trend in compensatory growth differed from that in wheat. Plants cut at a young age showed less growth in height, number of branches, number of pods and seeds (Fig 7A to 7D). Early damage appeared to be more detrimental in this species. Different extent of cutting at the pre-flowering stage showed compensatory growth negatively correlated to the extent of cutting (Fig 7E to 7H). In spite of regrowth, there was 40 to 80% loss in the seed number.

**Fig 8:**
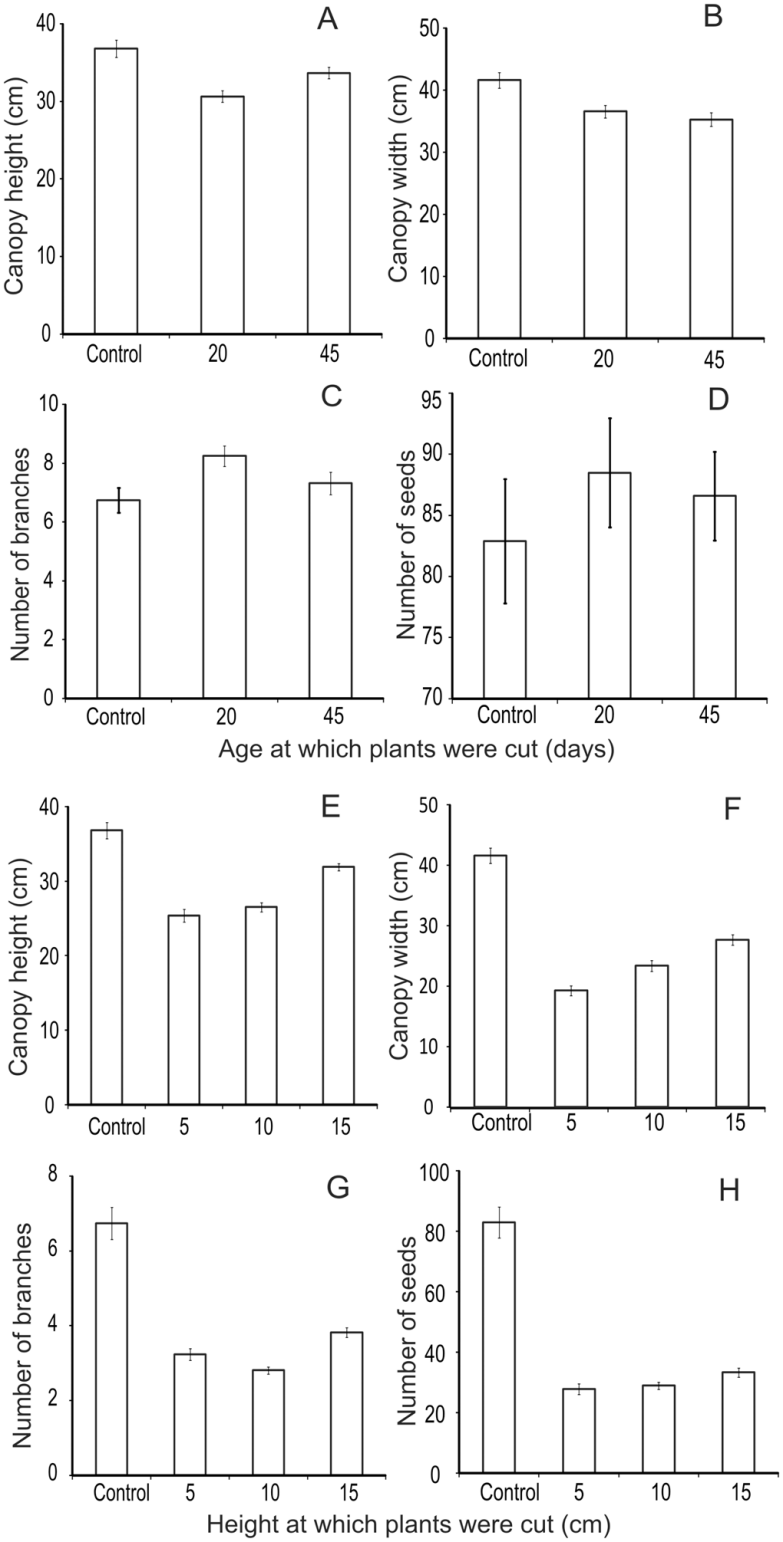
Compensatory growth after artificial herbivory at different ages in chickpea. A: canopy height, B: canopy width, C: number of branches and D: number of seeds in plants cut when 25 days old (n = 53) and 45 days old (n = 51) with control (n = 50). Compensatory growth after artificial herbivory at different heights at pre-flowering stage in chickpea. E: canopy height, F: canopy width, G: number of branches, H: number of seeds of plants cut at 5 (n = 50), 10 (n = 51), 15 (n = 54) with control (n = 50).

Artificial herbivory experiments on chickpea gave non-linear outcomes. Cutting at the age of 20 days led to greater branching ultimately resulting into increased number of seeds. Cutting at 45 days showed the same direction of effect but less pronounced (Fig 8A to 8D). This phenomenon is known to farmers and some farmers practice controlled plucking to increase the yield. However, cutting down beyond a threshold was counterproductive and decreased regrowth as well as seed formation. A yield deficit of up to 67% was noted on cutting down a plant to 5 at a pre-flowering stage (Fig 8E to 8H).

We did not perform artificial cutting in the case of rice, but did observe that in the unfenced and unguarded plot exposed to herbivory, the number of tillers bearing seed was about 26% less and the number of seeds per tiller were 32% less than the protected plot.

## Discussion

Many studies have pointed out the large difference between the amounts compensated and the perceived losses [32, 40–41]. However, attempts to make a reliable third party assessment of actual damage are few, most studies depending upon questionnaire surveys and oral impressionistic information. The uniqueness of our study lies in the attempts towards a first-hand assessment of damage using multiple methods.

We employed four different approaches to assess and compare crop damage in the study site. It is possible that each of the methods suffers from some flaw or shortfall. The net yield trends observed with distance from forest are likely to be affected by other factors. (a) It is likely that there is a trend with distance from the park in the fertility, water availability, irrigation facility or any other agriculture related property of soil. (b) Farmers close to the park tend to invest less in intensive agriculture owing to the risk of damage. It is possible to assess the two possibilities from the available data.

In experiments with fenced farms adjacent to the park, the yields observed were comparable to those at a distance of 5–6 km from the park. For rice, the protected farm yield was 21.66 Q/Ha, and for wheat 24.88 Q/Ha both being close to the regression yield expected at 5–6 km. Since giving protection alone could increase the yield to a level comparable to the highest yielding areas, soil fertility was an unlikely reason for the trend in yield with distance.

In contrast with (a) above, the possibility (b) was backed by some evidence. Farmers adjacent to the boundary hardly used chemical fertilizers, whereas at 10 km 90% farmers used more than one types of chemical fertilizers in combination. This trend is expected by optimization models of farmers’ economic (Watve et al manuscript under review). It is possible therefore, that farmers facing higher risk of herbivore damage invest relatively less in agricultural inputs and part of the reason for lower yields near the forest could be the trend in investment. In brief, greater accessibility to and frequency of visits by wild herbivores and farmers’ discouragement from investing in intensive agriculture appear to be responsible for the trend in grain yield (Watve et al manuscript under review). It should be noted that loss due to farmer’s disinvestment is indirectly caused by herbivory itself, but it is unlikely to be recorded during visual inspection of damage even if we assume the visual estimate to be accurate.

The poor correlation between visual estimate of damage and net loss in grain yield demonstrates that visual inspection is unable to reflect on realistic loss. If the ratio of the two estimates was fairly consistent it would have been possible to rely on visual estimates after applying certain correction factor. However, the distribution of the ratio of visual estimate to harvest based estimate was widespread and highly skewed. In addition, since the difference was in orders of magnitude, a small error in assessment would get amplified by orders of magnitude. This implies that visual damage estimations are both unreliable and grossly underestimating.

Results of simulated herbivory are important because a potential cause of mismatch between a visual estimation of damage and grain yield deficit is regrowth of plants after damage [33, 39, 42–43]. The vegetative parts of plants regrow to a considerable extent after herbivory [39]. There are claims of herbivory being beneficial for plants owing to stimulated regrowth [39, 42–43]. We observed some positive effect on chickpea after limited cutting. However, barring this exception the effects of herbivory on net yield were negative in our study. We suspect that some of the responses of different crop species to cutting are evolved life history optimization responses rather than the direct loss due to damage alone [44–45]. For example, chickpea may have evolved to respond to limited herbivory by preferring greater investment in reproduction. Rice on the other hand belongs to grasses that have substantial root biomass, which is long lived and can regrow in the following season. Therefore, on facing greater threat of seed predation it may strategically invest more in root biomass and less in seed production. Such life history strategies of crop species [45] may explain some of the observed patterns. These are interesting hypotheses that need to be pursued separately. Our limited goals did not permit us to pursue these lines of investigations.

Nevertheless, the artificial herbivory experiments demonstrated that although the plants showed the ability to regrow, there was a substantial loss in the yield. This is important since after damage within a few days the farm as a whole looks intact and green due to regrowth and therefore the damage may not be noticeable on visual inspection, but a substantial loss is incurred.

For crops other than rice, the regression of grain yield with distance estimated between 28 to 78% deficit adjacent to the park in comparison with the belt between 5–6 km. Experimental comparison of protected and unprotected farms revealed almost 100% loss for crops other than rice. In these experiments neither fencing nor guarding were employed. The farms neighboring the experimental farm had unfenced farms but they were being actively guarded by farmers every night. These guarded but unfenced farms incurred about 50±10% loss. The difference between the unfenced unguarded experimental farms and unfenced but guarded neighboring farms can be said to reflect the efficiency of manual guarding. By this calculation, manual guarding was able to save about 50% of loss. Compensatory growth studies after artificial herbivory revealed that although plants did give some grain yield, the net grain deficit in the experiment ranged between 40% and 70%. All the evidence converges to over 50% loss close to the park boundary. This matches the farmers’ perception closely and differs from the government records of damage.

There were two prominent mismatches in the independent assessments. The first one was that in the regression versus experimental estimation of damage in rice. Although the trend with distance was not consistent and therefore damage could not be calculated from the trend, the experimental plot showed substantial deficit in rice yield in the unprotected area as compared to the protected area. The deficit was unexpected since the observed frequency of raids was not very high. The difference can perhaps be due to post-harvest damage (e.g. depredation on stacks of harvested crop) by wild pig or differential strategic investment by the plants as discussed above.

The other major mismatch was that visual assessments always gave substantially lower estimates compared to all other methods. There are a multitude of possible reasons why visual assessment always gave underestimates. (i) The prevalent herbivore species in the study area do more of nibbling damage, which is less noticeable than trampling or uprooting type of damage. (ii) Not all types of damages are noticeable at the same time. For example, root or stem base chewing by wild boar leads to slow drying of the individual, which becomes noticeable after a few days. On the other hand nibbling the tips may be apparent after a careful look immediately after the damage, but the plants regrow soon and the damage becomes difficult to notice after a few days. By the current compensation protocols the inspection happens only once after filing a claim and there are variable delays between damage and inspection. Therefore it is difficult to notice all types of damages together in a single inspection. (iii) In the study area, the frequency of damage was high but the modal extent of damage per night small. The current inspection and compensation procedures are better suited for low frequency high extent damage. (iv) Since, the frequency of damaging raids is of the order of 0.3 per night, if every damaging raid is to be inspected and assessed there is a need to inspect every farm twice a week on an average. This puts an unrealistically large demand on competent and authorized personnel for inspection-validation work which appears impossible in the current set up. In reality no farm was inspected more than once in a crop season. Therefore in effect only a small part of damage was actually inspected (v) Farmers tend to disinvest from intensive agricultural practices when faced with high risk of damage (Watve et al, manuscript under review). This is unlikely to be recorded in visual assessment. (vi) Even if we assume that all actual losses are compensated realistically, the cost incurred in the protection measures is an additional burden that remains unaccounted for. (vii) Post-harvest damage, especially by wild pigs, is likely to be substantial for rice. This is generally not covered by the compensation procedures. Thus for a number of reasons the currently employed method of visual assessment is unable to make a realistic reflection of actual damage and thereby offer adequate compensation.

Previous research on crop raiding by wild animals in India is heavily biased towards damage by large herbivores such as elephants. In this case, the damaged area is measurable and the net loss is likely to be directly proportional to the fraction of the visibly damaged area. However, the case with smaller to medium sized herbivores that do not kill the plants is very different. It is possible that visual assessment of damage works for certain species of damaging animals, but fails completely for others. There is a need for alternative methods of damage estimation where a visual assessment fails. We suggest that it should be based on the grain yield or net produce at harvest rather than visually assessed vegetative loss. A model for compensation based on community data collection is suggested by Watve et al [34] that takes into account all possible flaws of such a system and an operating design that can overcome these flaws. This principle can be a potentially effective solution to make realistic damage compensations.

It is in the interest of PAs to address the conflict problems realistically to avoid growing resentment that can potentially mount over time to explode at some stage. The problem needs to be addressed at multiple levels including measures to reduce the damage, encouraging alternative crop species non-palatable to herbivores, alternative livelihood along with realistic damage compensation [27, 46–48]. The main concern is prevention of social damage more than economic damage. Anticipatory and preventive solutions need to be implemented rather than looking for remedies after a major episode of unrest [27, 47].
